# Inter-sexual and inter-generation differences in dispersal of a bivoltine butterfly

**DOI:** 10.1038/s41598-021-90572-1

**Published:** 2021-05-26

**Authors:** Elisa Plazio, Piotr Nowicki

**Affiliations:** Institute of Environmental Sciences, Jagiellonian University, Gronostajowa 7, 30–387 Kraków, Poland

**Keywords:** Ecology, Evolution, Zoology, Ecology

## Abstract

In organisms with discrete generations such as most insects, life-history traits including dispersal abilities often vary between generations. In particular, density-dependent differences in dispersal of bi- and multivoltine species may be expected because subsequent generations are usually characterized by a drastic increase in individual abundance. We investigated the inter-sexual and inter-generation differences in dispersal of a bivoltine butterfly, *Lycaena helle*, testing the following hypotheses: (1) male emigration is higher in spring generation, as males are prone to leave their natal habitat patches when the density of mating partners is low; (2) female emigration is higher in summer generation, when it helps to reduce intraspecific competition between offspring. The outcome of our analyses of dispersal parameters showed that females of the summer generation emigrated from their natal patches considerably more often than those of the spring generation, whereas an opposite trend was detected in males. These findings offer a novel perspective for our understanding of the advantages of voltinism for metapopulation functioning. The spring generation dispersal mainly improves the random mating opportunities favoured by the increase in male emigration. In turn, the dispersal of females of the summer generation appears the key to long-term metapopulation persistence.

## Introduction

In organisms with rapid life cycles and discrete generations, such as most insects living in temperate and cold zones, the responses to climatic variability include alteration of phenology such as shifts of activity towards an earlier time of year in warmer climates^1,2^, prolonging or shortening the activity period^3,4^ and changing the number of generations (voltinism) per season^5,6^. Based on the number of generations within a year, organisms are categorized into univoltine if represented by one generation per year, bivoltine with two generations per year, or multivoltine in the case that they have more than two generations per year^7^. There may be also cases of organisms whose generation time is longer than one year and such species are defined semivoltine.

Voltinism is determined under both genetic and environmental control and, as for its environmental component, it depends on photoperiod and temperature^8^. Generally, the number of generations per year of an insect species decreases with increasing latitude or altitude, and this is often accompanied with changes in developmental phase and duration of the diapause (i.e., the state of arrested growth or reproduction that is typical for many hibernating or aestivating arthropods; cf. Lees^9^), which could eventually even lead to morphological changes, e.g., in body size of adults^7^. Therefore, populations of the same species can be univoltine at higher altitudes and latitudes and bi- or multivoltine in lowlands and at lower latitudes. Information about voltinism, i.e., the number of generations completed within one year, is needed in order to understand how species have adapted to environmental conditions in different regions of the globe.

The increase in the number of generations per season is the final step of a series of events occurring when organisms experience a warming of climatic conditions. First, the local increase in temperature, if constantly maintained over years, may cause warming of the previously harsh periods of the year, which extends the season characterized by conditions favourable for the development and reproduction of organisms. In the case of insects, this may lead to an earlier onset of the imago occurrence period. Consequently, individuals of the first generation reproduce earlier. The favourable climatic conditions (i.e., higher temperatures) that the new generation of larvae find after egg hatching leads to their rapid growth and development, which ultimately affects voltinism; namely more individuals develop in a subsequent generation within the same year^8^.

Many multivoltine organisms show inter-generation variation in mobility^10^. Such variation often depends on morphological and biomechanical variation among subsequent generations, where at least one generation is characterized by morphological constraints, making it less mobile. A classic example is the case of many non-heteropteran Hemiptera (e.g. aphids), but also Heteroptera and Orthoptera species, which alternate between sedentary generations with shortened or absent wings and dispersive generations with fully developed wings^10^. A certain degree of inter-generation biomechanical polymorphism related to variation in mobility, although devoid of macroscopic morphological handicaps such as complete lack of wings, was also found in butterflies. The examples include *Pararge aegeria*^11^ and *Araschina levana*^12^, in which biomechanical polymorphism results in fast and energetically demanding flights of the first generation and long-endurance flights in the second one. Besides, in the well-known multivoltine migrants such as *Danaus plexippus*^13^, *Vanessa atalanta*^14^, and *Vanessa cardui*^15^, differences in mobility among generations are perceived as a consequence of the alternation between dispersive and reproductive sedentary broods. For other butterflies, like *Pieris* spp ^16^. and *Aglais urticae*^17^, inter-generation differences in mobility are related to variation in abundance and regional distribution of subsequent generations.

The density of conspecifics has been suggested by a number of previous studies as one of the main drivers affecting dispersal dynamics, with both positive and negative effects, leading to an increase or decrease in emigration rates^18–21^. A high density of conspecifics may additionally have a negative impact on the habitat quality. In the case of butterflies, the habitat quality refers primarily to the availability of the most important resource, namely the larval foodplants, which are particularly important for ovipositing females. With high conspecific densities, emigration propensity of females should increase, because by laying eggs in other (potentially less crowded) habitat patches they reduce the risk of their offspring competing for the foodplants, thus increasing offspring survival^21,22^.

On the other hand, a high density of individuals, and hence a high number of females within a given area, gives males the opportunity to maximize the number of mating occasions^23^. Therefore, under such circumstances, males should be expected to be less prone to leave their natal patches. The situation is reversed in the case of low density of individuals when it should be more beneficial for males to search for new patches with higher availability of mating partners.

Bi- or multivoltine species appear to be convenient models to study the effect of conspecific density on dispersal dynamics because subsequent generations are usually characterized by drastic differences in density of individuals. The sizes of local populations typically increase several times in subsequent generations^8^. This stems from the development of larvae in extremely different weather conditions which critically affects their survival^8^. Hence, an investigation into inter-generation variation in dispersal may highlight the relationship between individual density and dispersal in both sexes. Nevertheless, density dependence of dispersal and sex-biases in this respect have so far been investigated mainly by analysing inter-annual patterns^20,24–26^ (but see Plazio et al*.*^21^ on the variation of emigration propensity within the flight season).

Following the above rationale, in the present study we focused on the intersexual differences in dispersal between generations of a bivoltine butterfly *Lycaena helle,* having a spring generation characterized by low density of individuals and a summer generation with high density of individuals. We tested the following hypotheses: (1) male emigration is higher in the spring generation, as males are prone to leave their natal habitat patches when the density of mating partners is low; (2) female emigration is higher in the summer generation, when it helps to reduce intraspecific competition between offspring.

## Materials and methods

### Study species

*L. helle* is a boreal species with the occurrence range extending from Central Europe, Scandinavia, and Russia to the Amur Region and Mongolia^27^. In Central Europe it is a postglacial relict, present in isolated univoltine populations mostly restricted to mountain areas^28^, whereas bivoltine populations are present also at lower altitudes in Eastern Europe, Mongolia, some areas in the Ardennes, and parts of Germany and Poland^27^. In our study region (southern Poland), the spring generation begins at the beginning of May and usually lasts until the beginning of June, while the summer generation appears at the beginning of July and lasts until early August. The species shows a marked sexual dimorphism. The upperside of male wings is characterized by a strong violet iridescence that is, instead, restricted to a submarginal band of iridescent ocelli in females. The caterpillars are monophagous, feeding on *Polygonum bistorta* in our study area^29^, and thus the occurrence of the species is limited to meadows with this foodplant. The foodplant is also used as the primary nectar source by imagoes. In addition, adults were found feeding on a variety of other nectar plants, with a preference for *Ranunculus* spp. and *Cardamine* spp.^27,30^. Being associated with very fragmented habitats with a patchy distribution of the foodplant, *L. helle* typically occurs in classic metapopulation systems with discrete local populations and their habitat patches being easy to define^29^. Despite being generally regarded as sedentary, it may sporadically perform long-distance movements^31^. The species currently represents one of the most endangered butterflies in Europe, listed in the European Red Data Book as well as in the Annexes II and IV of the Habitats Directive^32,33^. Deterioration of meadow habitats caused by land drainage and abandonment of traditional management as well as their increasing fragmentation have been suggested as the main reasons for the decline of the species detected in recent decades^27,34^.

### Study area

The study was conducted within a large meadow complex (ca. 800 ha) located in the Vistula River valley on the south-west outskirts of the city of Kraków, southern Poland (50º01’N, 19º54’E). The complex consists of *Molinion* wet meadows and lowland hay meadows representing the *Arrhenatherion elatioris* community, with occasional *Festuco-Brometea* xerothermic grasslands occupying elevated land fragments. The meadows are mostly abandoned, with a small percentage of the area being mowed annually. Since 2011 a large part of the area has been protected in the Natura 2000 site “Dębnicko-Tyniecki Obszar Łąkowy” (PLH 120065), established for the conservation of four butterfly species listed in the Annex II of the Habitats Directive 92/43/EEC: *Maculinea teleius*, *M. nausithous*, *L. helle*, and *L. dispar*^35^.

The entire study system includes 11 *P. bistorta* patches, of variable area and isolation, inhabited by local populations of *L. helle* (see the map in Nabielec and Nowicki^29^). The area of individual patches ranges from 0.26 to 2.77 ha. The degree of isolation corresponding to the distance of a given patch from the nearest other patch ranges from 200 to 1500 m. The foodplant patches form a classic metapopulation, with local *L. helle* populations acting as independent demographic units^29^. The investigated patches are free from direct human impacts, either negative (e.g., habitat destruction) or positive ones (e.g., targeted conservation management). All the habitat patches were mapped with high-precision GPS and their spatial parameters (i.e., location and area of each habitat patch) were subsequently derived in GIS software ArcGIS and Idris for Windows.

### Field sampling

Our study in 2018 and 2019 involved a large fraction of the system, with 8 foodplant patches surveyed. The field surveys, based on intensive mark-release-recapture (MRR) sampling^36^, were carried out in four campaigns conducted in two consecutive years (2018: from May 14 to June 6, and from June 29 to July 31; 2019: from May 8 to June 6, and from June 28 to July 30) according to the occurrence of imagoes in the two generations of *L. helle* in our study area. Butterflies were captured using a butterfly net and each individual was marked with a waterproof marker (Staedtler Lumocolor 313) by writing a unique number on the underside of its hind wing. Marking did not adversely affect the condition of the butterflies. After marking, the specimens were immediately released at the place of their capture. For each individual, the assigned number along with the sex, catch time, and code of the habitat patch was recorded. In accordance with the flight activity of *L. helle* adults, the MRR sampling was performed between 9:00 and 17:00 on a daily basis in appropriate weather conditions (with few gaps due to rainfall, strong wind, or air temperature < 15 °C). To ensure adequate sampling effort and fairly uniform capture probability, the time spent in each patch was adjusted to its area and the abundance of butterflies flying (based on the experience from earlier years), varying from 1.5 to 4 person-hours per day. The order in which patches were visited each day was randomly changed throughout the season so as to avoid sampling the same patch at similar hours every day. This procedure allowed to avert potential biases in the frequencies of butterfly movements recorded between particular patches due to the fact that butterfly activity may be influenced by daily weather patterns, especially temperature (cf. Cerrato et al.^37^). The protocol of the methodological procedure described above was identical for the four MRR campaigns.

### Estimation of dispersal parameters

The parameters of dispersal within the investigated metapopulation were estimated based on the MRR data collected, separately for each sex, generation (spring or summer), and year. The estimates of dispersal parameters were derived with the help of the Virtual Migration (VM) model which represents a well-established standard for analysing dispersal using MRR data^38^. The model requires the following assumptions: (i) individuals inhabit a network of discrete habitat patches with different size and connectivity, (ii) at least 7 patches have been sampled with MRR, (iii) the sampling has involved 10 or more capture occasions, and (iv) spatial information is available for both the sampled patches as well as those that were not sampled because their presence affects the connectivity of the former group^26,38^. All these underlying assumptions were clearly met in our study.

The basic parameters estimated by the model include: mortality in habitat patches (*μ*), emigration propensity (*η*) defined as daily emigration rate scaled to 1-ha patch, emigration scaling with natal patch area (*ξ*_*em*_), immigration scaling with target patch area (*ζ*_*im*_), scaling of mortality during dispersal with natal patch connectivity (*λ*), and distance dependence of dispersal (*α*). The model assumes that an individual survives in any habitat patch with a probability of *ϕ* until the end of the unit of time (in the case of butterflies the unit is usually scaled to one day) or until emigrating from the patch. The dispersal-independent mortality in each habitat patch (*μ*) is therefore 1–*ϕ*. As an independent evaluation of this parameter with the Cormack-Jolly-Seber models^39^ revealed no intersexual differences, we assumed a uniform value for males and females in each season in order to increase the precision of further parameters derived with the VM model, following the approach already used in previous works^21,26^. The mean adult life-span can be estimated from the survival rate^40^ as (1 − *ϕ*)^−1^ − 0.5, and therefore derived directly from the estimates of dispersal-independent mortality in each habitat patch (*μ*) as 1/*μ* − 0.5.

The emigration rate *ε*_*j*_ from a specific patch *j* is modelled as a function of patch area:

1 $$ \varepsilon_{j} = \eta A_{j}^{ - \xi em} $$

where *η* defines emigration propensity, and the emigration scaling parameter (*ξ*_*em*_) reflects the steepness of the negative power relationship of actual emigration rate with patch area (*A*_*j*_). The rationale for this negative relationship is that with decreasing patch area the individuals are more likely to reach the edges of their natal patch, and subsequently leave it^41^.

The survival of dispersing emigrants from a patch *j* (*φ*_*mj*_) is assumed to increase with the patch connectivity *S*_*j*_ (i.e. the inverse index of its spatial isolation), with a relation described by a sigmoid function:

2 $$ \phi_{mj} = \frac{{S_{j}^{2} }}{{\lambda + S_{j}^{2} }} $$

where the scaling parameter *λ* represents the connectivity value at which half of the dispersers successfully reach other patches. The connectivity index is calculated as:

3 $$ S_{j} = \mathop \sum \limits_{k \ne j} \exp ( - \alpha d_{jk } )A_{k}^{\zeta im} $$

where *d*_*jk*_ is the Euclidean distance between patches, and *A*_*k*_ refers to the target patch area, with which the immigration rate is linked through the scaling parameter *ζ*_*im*_. The parameter *ζ*_*im*_ assumes positive values as the chance of immigration positively depends on the area of the target patch. This dependence is explained by the fact that it is much easier to reach large patches in close proximity to the natal patch^41^. Finally, *α* represents the scaling of distance-dependence of dispersal, i.e., the coefficient of the kernel describing the probability of dispersal at a given distance.

The dispersal-related mortality scaling (*λ*) and distance-dependence of dispersal (*α*) can be converted into the mean level of mortality experienced by dispersers and the mean distance they cover, respectively. The mean dispersal distance is calculated as 1/*α*.

The maximum likelihood (ML) values and the 95% confidence intervals (95% CIs) for the VM model parameters were obtained using respectively the VM2 and VMSIM programs^38^. We tested the significance of the differences between the parameters obtained for both sexes in both generations and years by comparing their 95% CIs. The difference between a pair of parameters should be considered significant at *P* < 0.05 if the ML value of one parameter falls outside the 95% CIs of the other parameter and vice versa^42^.

### Ethics approval

Experiments comply with current laws of Poland where they were performed. The fieldwork was conducted with the proper permission based on the conditions of granted exemptions (Polish General Directorate for Environmental Protection). Experiments also comply with the ARRIVE guidelines (PLoS Bio 8(6), e1000412,2010).

## Results

Over two years of the study we recorded 3211 individuals of *L. helle*, with a population in the second year almost three-times larger than in the previous year of the study (Table 1). As expected, the summer generation was several times more abundant than the spring one in both years. The sex ratio of captured individuals was consistently male-biased (Table 1).

**Table 1:** Sample sizes of *L. helle* captured through mark-capture surveys in the Dębnicko-Tyniecki Obszar Łąkowy, southern Poland.

Year	Spring generation				Summer generation			
	Males	Females	Total	Sex ratio (m/f)	Males	Females	Total	Sex ratio (m/f)
2018	70	44	114	1.60	408	305	713	1.33
2019	148	83	231	1.78	1331	822	2153	1.62

The analyses conducted with the VM model using the data collected through the MRR campaigns of 2018 and 2019 showed an overall similar pattern. An overview of the obtained dispersal parameters is given below, while a detailed list of all the estimate values, together with their 95% CIs, is presented in Tables 2 and 3.

**Table 2:** Dispersal parameter estimates (with their 95% confidence intervals in parentheses) obtained for *L. helle* butterflies with the Virtual Migration model in 2018. Following the outcome of the Cormack-Jolly-Seber models, no inter-sexual difference in mortality in habitat patches, and consequently also in average adult life-span, was assumed (see the Methods section for details).

Parameter	Females		Males	
	Spring generation	Summer generation	Spring generation	Summer generation
Mortality in habitat patches (*μ*)	0.1520 (0.1130–0.1957)	0.2231 (0.2015–0.2466)	0.1520 (0.1130–0.1957)	0.2231 (0.2015–0.2466)
Emigration propensity (*η*)	0.0167 (0.0031–0.0397)	0.1189 (0.0788–0.1657)	0.0708 (0.0381–0.1149)	0.0277 (0.0161–0.0441)
Emigration scaling with natal patch area (*ξ*_*em*_)	− 0.7139 (− 1.9065–0)	− 0.0476 (‒0.3775–0)	0 (− 0.5469–0)	− 0.0321 (‒0.5127–0)
Immigration scaling with target patch area (*ζ*_*im*_)	0 (0–1.6692)	0.2482 (0–0.7996)	0 (0.–0.4357)	0 (0–0.4600)
Dispersal mortality scaling with natal patch connectivity (*λ*)	0.7041 (0–3.0172)	0.4596 (0.1352–0.8480)	0.8724 (0–2.7895)	0.0101 (0–1.4493)
Distance dependence of dispersal (*α*)	2.7093 (0–4.2291)	4.5060 (3.0868–5.8623)	1.2392 (0–2.5460)	1.9996 (0.3514–4.3125)
Mean dispersal distance (1/*α*) [m]	369 (236–∞)	222 (171–324)	807 (393–∞)	500 (232–2845)
Average life-span (1/*μ*‒0.5) [days]	6.08 (4.61–8.35)	3.98 (3.55–4.46)	6.08 (4.61–8.35)	3.98 (3.55–4.46)

**Table 3:** Dispersal parameter estimates (with their 95% confidence intervals in parentheses) obtained for *L. helle* butterflies with the Virtual Migration model in 2019. Following the outcome of the Cormack-Jolly-Seber models, no inter-sexual difference in mortality in habitat patches, and consequently also in average adult life-span, was assumed (see the Methods section for details).

Parameter	Females		Males	
	Spring generation	Summer generation	Spring generation	Summer generation
Mortality in habitat patches (*μ*)	0.1915 (0.1558–0.2308)	0.1873 (0.1734–0.2016)	0.1915 (0.1558–0.2308)	0.1873 (0.1734–0.2016)
Emigration propensity (*η*)	0.0567 (0.0249–0.1083)	0.3155 (0.2686–0.3686)	0.1559 (0.0847–0.2512)	0.0662 (0.0507–0.0849)
Emigration scaling with natal patch area (*ξ*_*em*_)	− 0.1262 (–0.8078–0)	− 0.0976 (‒0.2238–0)	0 (− 0.4029–0)	− 0.2456 (− 0.4748–0)
Immigration scaling with target patch area (*ζ*_*im*_)	0.4031 (0–1.4788)	0.0524 (0–0.2370)	0 (0.–0.3273)	0 (0–0.2957)
Dispersal mortality scaling with natal patch connectivity (*λ*)	0 (0–3.935)	0 (0–0.4269)	0.9257 (0–1.7914)	0.4022 (0–0.7585)
Distance dependence of dispersal (*α*)	1.3115 (0–4.4841)	1.3295 (0.7699–1.9699)	3.1180 (0–4.6790)	2.8402 (1.9248–3.5694)
Mean dispersal distance (1/*α*) [m]	762 (223–∞)	752 (508–1299)	321 (214–∞)	352 (280–520)
Average life-span (1/*μ*‒0.5) [days]	4.72 (3.83–5.92)	4.84 (4.46–5.27)	4.72 (3.83–5.92)	4.84 (4.46–5.27)

In 2018, the mortality in habitat patches (*μ*) corresponded to approximately 15% of individuals of the spring generation dying per day while, in the summer generation, it reached approximately 22% (Table 2). In 2019, the mortality within patches was approximately 19% for both generations (Table 3). The average butterfly life-span varied approximately between 4 and 6 days (Tables 2, 3).

**Figure 1:**
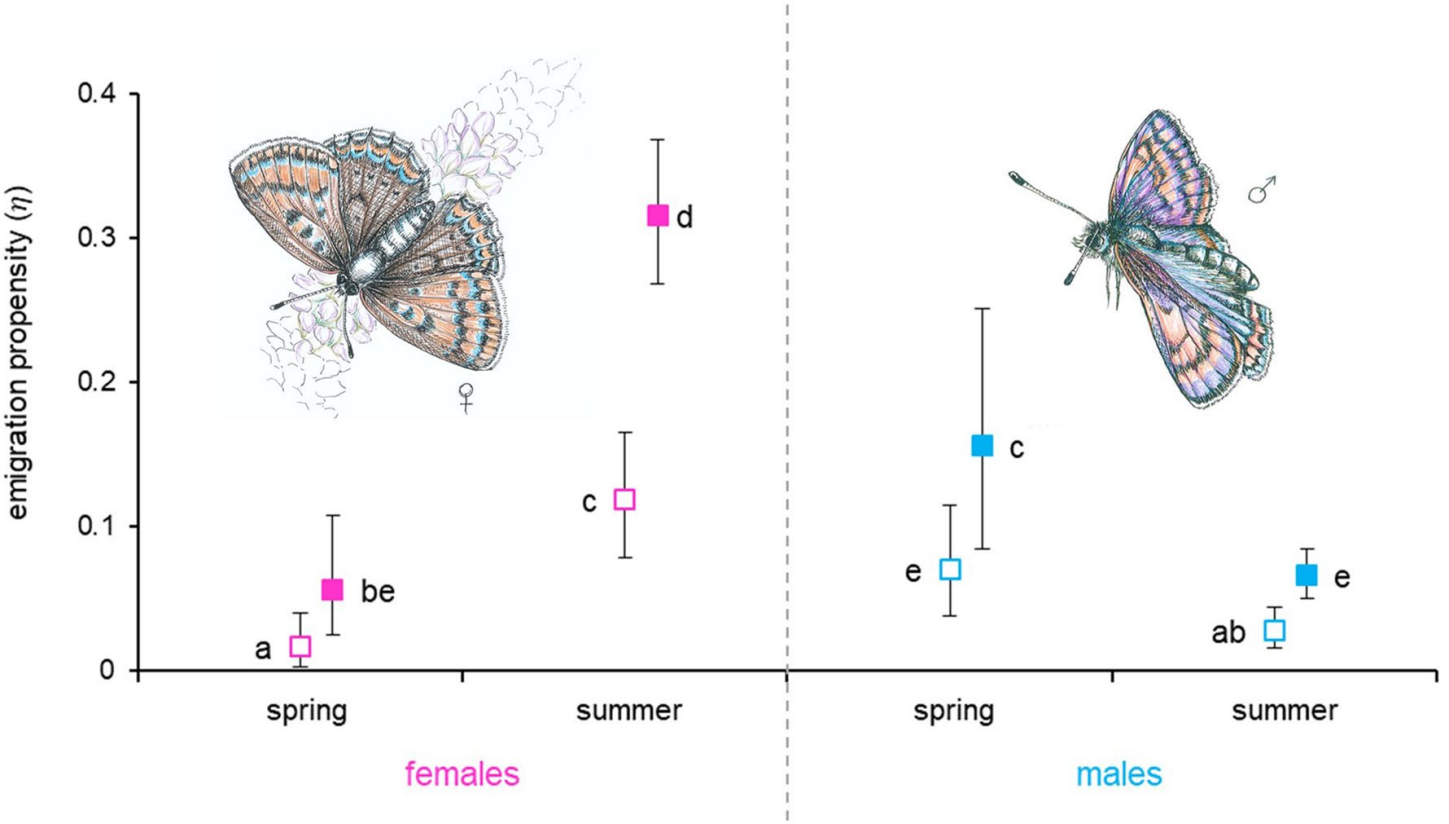
Between-season variation in emigration propensity of *L. helle* adults of both sexes in 2018 (empty symbols) and 2019 (solid symbols). The estimate values are presented with their 95% confidence intervals; the values statistically different (at *P* < 0.05) from each other are marked with different alphabet letters.

The emigration propensity (*η*) of both sexes statistically differed between spring and summer generation, with an almost identical pattern in both years of the study, despite the fact that in 2019 the parameter *η* values for both sexes and generations were higher than in the previous year, characterized by a lower population density. Overall emigration propensity of females was greater than for males (Fig. 1, Tables 2, 3). Most importantly, as expected, females belonging to the summer generation emigrated significantly more often from their habitat patch than those of the spring generation, with the parameter *η* values growing ca. 6–7 times between spring and summer in both years (Fig. 1, Tables 2, 3). The pattern was in turn the opposite for males, with individuals belonging to the spring generation significantly more prone to emigrate, although in this case, inter-generation variation was much lower than females with the spring emigration propensity being ca. 2–3 times higher (Fig. 1, Tables 2, 3).

The estimates of emigration scaling with the natal patch area (*ξ*_*em*_) were invariably very close or equal to zero, except for females of the spring generation in 2018 (Table 2). Nevertheless, the estimated value did not significantly differ from zero, indicating absolutely no evidence for any dependence of emigration on patch size. Likewise, we found no indication of the effect of target patch area on immigration, with the immigration scaling parameter (*ζ*_*im*_) values being typically very low and never significantly different from zero (Tables 2, 3).

No clear patterns were found in the dependence of mortality during dispersal on natal patch connectivity (*λ*), but again the values of this parameter were not significantly different from 0 in most cases, which indicates very low (if any) disperser mortality. A single exception were females in summer 2018, for which significantly non-zero dispersal-related mortality was detected, but still it was estimated at a relatively low level with a few percent of individuals leaving their habitat patches.

**Figure 2:**
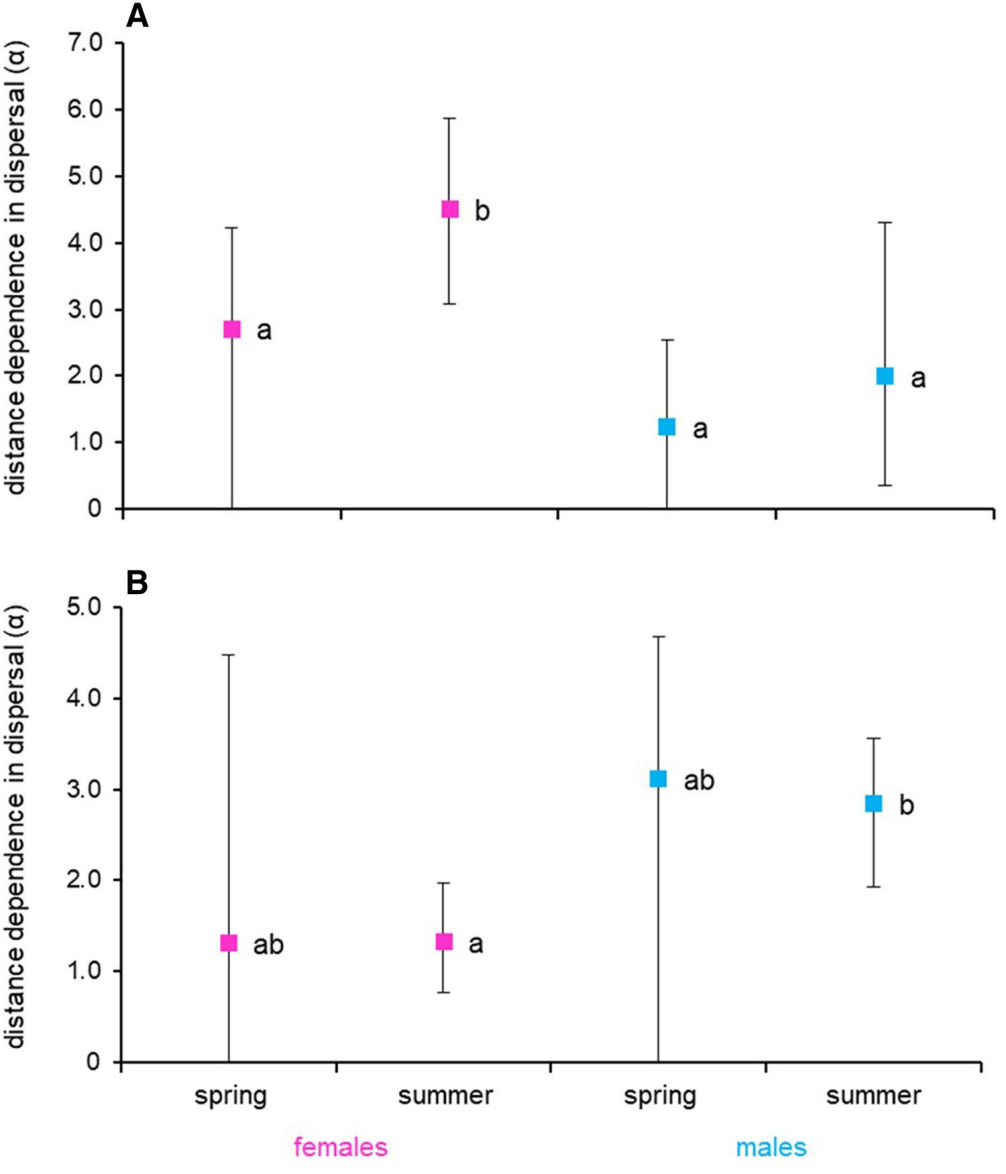
Between-season variation in distance dependence of dispersal in *L. helle* adults of both sexes in 2018 (**A**) and 2019 (**B**). The estimate values are presented with their 95% confidence intervals; the values statistically different (at *P* < 0.05) from each other are marked with different alphabet letters.

In 2018 significant differences in the distance dependence of dispersal (*α*) were found between females of the two generations, with spring generation individuals covering longer distances, whereas no such differences were found in the case of males (Table 2, Fig. 2a). Compared with females, males of both generations travelled longer distances in 2018. Surprisingly, the pattern in 2019 was the opposite, as females of both generations travelled longer distances than males (Table 3, Fig. 2b). Besides, the results of 2019 showed almost no difference between the two generations in the average distance travelled.

## Discussion

The drastic differences in density of individuals between generations in the *L. helle* metapopulation allowed us to study inter-generational dispersal dynamics. We also found metapopulation size varied by about three times between 2018 and 2019. Such inter-annual fluctuations in (meta)population size are typical for butterflies^43^, but in both years the inter-generation pattern was similar, with the second (summer) generation being much more abundant than the first (spring) one. Such a difference most likely stems from the fact that a large fraction of wintering pupae from the summer generation do not survive due to unfavourable weather conditions in winter^29^. This is a common phenomenon in bi- and multivoltine species^8^.

Due to this strong difference in the abundance of the two subsequent generations, one may expect an effect on individual traits. Intra-generation variation in several morphological and behavioural traits was detected by previous studies, including many studies on butterflies^8^. In the case of butterflies, for which flight is one of the main activities affecting individual fitness, it is reasonable to expect variation in dispersal propensity between individuals belonging to different generations due to differences in the density of conspecifics. The effect of conspecific density on dispersal propensity is well grounded in the theory that predicts higher emigration whenever the population experiences stronger competition^44^. Intersexual differences in density-dependent dispersal was already detected by a number of previous studies^21,45–48^, where population density was usually positively associated with female emigration, while it had a negative effect on male emigration.

In agreement with this scenario, we expected that male emigration should be higher in the spring generation, as males are prone to leave their natal habitat patches when the density of mating partners is low, while female emigration should be higher in the summer generation when it helps to reduce intraspecific competition between offspring. The results obtained through the analyses of dispersal parameters confirmed our hypotheses. Indeed, females of the summer generation, characterized by high density of individuals, tended to emigrate more frequently from their habitat patches. Previous studies suggested different causes of intersexual differences in density-dependent dispersal^49,50^. The majority of them agreed that density-dependent dispersal exists as a consequence of social interactions among individuals of the local population^45,47,48^. In many butterfly species, when the density of conspecific individuals is high, females may leave the natal patch due to harassment exerted on them by males looking for a mate. In turn, in the case of low density of individuals and specifically low density of males, the effect of harassment on females is negligible^45,46,48^. However, other studies suggested that sexual harassment-driven dispersal of females appears to be a relatively rare phenomenon when compared with dispersal performed to reduce intraspecific competition for resources experienced by offspring^20,21,50^. In this alternative scenario, a primary driver of female dispersal is their pursuit for the optimal place for oviposition. Specifically, the density of females may affect the habitat quality in a given area in terms of abundance of available foodplants, which determines the habitat carrying capacity for ovipositing females. Therefore, it should be expected that emigration will increase when the density of individuals exceeds the local capacity of the habitat^51,52^. Specifically for butterflies, this may lead females to leave their natal patches in case of improper quality or scarcity of foodplants in order to reduce their offspring competition for resources^21,47^. Leaving a natal habitat patch with a high density of conspecifics may thus be particularly beneficial for females, as it gives them a chance to lay eggs in less crowded habitats. Moreover, this behaviour of females also reduces the risk of inbreeding among offspring^53^.

Conversely, lower male emigration is expected from a population with high density of individuals^23,54^. This tendency may be explained by the fact that males aim to maximize the number of mating occasions during their lifetime in order to increase their evolutionary success^21,24,55^. The abundance of available females is the key factor determining the habitat quality for males. Our results confirm the above rationale, as emigration propensity was consistently significantly higher in males from the spring generation, characterized by low density of individuals. With an increase in female density in summer generation, the competition between males for access to mating partners decreased, apparently leading to a decrease in male emigration.

However, our results suggest that dispersal in the investigated *L. helle* metapopulation is not solely driven by conspecific density per se, specifically in the case of males. First of all, the emigration propensity of males in the summer generation of 2019 turned out very similar to that of males in the spring generation of 2018, despite much higher (by over an order of magnitude) female abundance in the former period. This may be because male decision to emigrate is likely to be influenced not only by female availability but also by the sex ratio, which was strongly male-biased in summer 2019, thus increasing competition for females. The outcomes of the present study also provide interesting insights into the pattern of distances travelled by the butterflies of both sexes. Specifically, in 2018 males of the spring generation performed longer movements than females and males of the summer generation, while in 2019 the situation changed, with longer distances covered by females. This may be explained by the fact that in the spring of 2018 (characterised by several times lower butterfly numbers than in the following year) the extreme scarcity of flying females forced males to travel longer distances in order to find a mate. For the same reason, females had to fly relatively short distances before finding a foodplant free from conspecific eggs already laid.

Apart from density-dependent effects on dispersal, our results highlight a clear difference in mobility of the two generations of *L. helle,* thus giving us the opportunity to consider a differential functional role of the two generations of bivoltine butterflies. A functional explanation of inter-generation differences in butterfly dispersal was given by Fric and Konvicka^12^, who hypothesized that individuals of the earlier generation, normally less abundant, tend to remain at the site of their emergence, mainly performing inter-patch routine movements, whereas those of the later generation tend to disperse. In *A. levana* demography modelling indeed revealed a higher mobility of the second (summer) generation^56^. Similarly, in *P. aegeria* morphological differences between early-spring, late-spring, and summer generations of the species have been detected and suggested to influence their flight performance^11^. In particular, early-spring males have the lowest values of relative thorax mass but the highest values of wing loading among males. In addition, late-spring males have larger relative thorax mass, wing loading, and aspect ratio than summer males. Such morphological differences undoubtedly indicate three different levels of energy investment in morphological features relevant for flight performance in three subsequent generations of the species which are likely to translate into differences in their mobility. Among females, late-spring individuals are the largest and heaviest and this characteristic is supposed to influence their flight performance as well^57^. Moreover, in their study on *A. levana,* Fric and Konvicka^12^ clearly linked behavioural differences in flight with morphological differences, suggesting that adults of summer generation, having a heavier thorax, lower abdomen to body mass ratio, larger wing area, less pointed wings, and lower wing loading than spring-generation individuals, are perfectly designed for long-endurance flights.

Although only a slight variation in wing size has been detected between generations of *L. helle,* with individuals belonging to the first generation having marginally bigger wings^27^ and hardly any inter-sexual or inter-generation differences in the body mass^58^, the inter-generation differences in mobility of the two sexes revealed by our analyses provide clear evidence of the different role that the two generations play in metapopulation functioning. This may offer a novel perspective for our understanding of the advantages of voltinism for metapopulation persistence. Interpreting our results from this perspective, it is reasonable to conclude that the spring generation dispersal of *L. helle* mainly improves the random mating opportunities favoured by an increase in male emigration whenever the density of females in the natal patch is low. On the other hand, the dispersal of females of the summer generation appears the key to the long-term persistence of the focal metapopulation. This is because while inter-patch movements by both sexes can contribute to gene transfer^59,60^, only female (post-mating) dispersal enables effective (re)colonisation of vacant habitat patches^61^. Since *L. helle* is considered a relatively poor colonizer of discrete habitat fragments^34,62,63^, female inter-patch movements, performed predominantly in summer when not only female numbers but also their emigration rates are several times higher, are fundamental for ensuring the species presence in fragmented landscapes. Therefore, any disturbance to female movements in summer season, e.g., unfavourable weather conditions reducing life expectancy of flying adults, would drastically limit colonisation potential, which determines metapopulation persistence^64^.

## Data Availability

The datasets generated during and/or analysed during the current study are available from the corresponding author on reasonable request.
